# The Circadian Clock Improves Fitness in the Fruit Fly, *Drosophila melanogaster*

**DOI:** 10.3389/fphys.2019.01374

**Published:** 2019-11-01

**Authors:** Melanie Horn, Oliver Mitesser, Thomas Hovestadt, Taishi Yoshii, Dirk Rieger, Charlotte Helfrich-Förster

**Affiliations:** ^1^Neurobiology and Genetics, Theodor-Boveri Institute, Biocenter, Julius-Maximilians University Würzburg, Würzburg, Germany; ^2^Theoretical Evolutionary Ecology Group, Biocenter, Department of Animal Ecology and Tropical Biology, Julius-Maximilians University Würzburg, Würzburg, Germany; ^3^Graduate School of Natural Science and Technology, Okayama University, Okayama, Japan

**Keywords:** competition, *period* mutants, resonance theory, mating preference, fertility

## Abstract

It is assumed that a properly timed circadian clock enhances fitness, but only few studies have truly demonstrated this in animals. We raised each of the three classical *Drosophila period* mutants for >50 generations in the laboratory in competition with wildtype flies. The populations were either kept under a conventional 24-h day or under cycles that matched the mutant’s natural cycle, i.e., a 19-h day in the case of *per*^*s*^ mutants and a 29-h day for *per*^*l*^ mutants. The arrhythmic *per*^0^ mutants were grown together with wildtype flies under constant light that renders wildtype flies similar arrhythmic as the mutants. In addition, the mutants had to compete with wildtype flies for two summers in two consecutive years under outdoor conditions. We found that wildtype flies quickly outcompeted the mutant flies under the 24-h laboratory day and under outdoor conditions, but *per*^*l*^ mutants persisted and even outnumbered the wildtype flies under the 29-h day in the laboratory. In contrast, *per*^*s*^ and *per*^0^ mutants did not win against wildtype flies under the 19-h day and constant light, respectively. Our results demonstrate that wildtype flies have a clear fitness advantage in terms of fertility and offspring survival over the *period* mutants and – as revealed for *per*^*l*^ mutants – this advantage appears maximal when the endogenous period resonates with the period of the environment. However, the experiments indicate that *per*^*l*^ and *per*^*s*^ persist at low frequencies in the population even under the 24-h day. This may be a consequence of a certain mating preference of wildtype and heterozygous females for mutant males and time differences in activity patterns between wildtype and mutants.

## Introduction

One of the main tasks of a circadian clock is to time animal daily activity and sleep to the right time of the day. The activity-sleep patterns of clock mutants without a circadian clock or with a clock running too fast or too slow are usually different from wild-type animals as has been demonstrated for rodents (Ralph and Menaker, 1988; van der Horst et al., 1999; Pendergast et al., 2010), fruit flies (Konopka and Benzer, 1971; Hamblen-Coyle et al., 1992; Hamblen et al., 1998) and humans (Toh et al., 2001; Patke et al., 2017). While animals without functional clock appear to merely respond to the daily light-dark changes, animals with too fast clocks have early activity-sleep phases and animals with too slow clocks have late activity phases, respectively (Hamblen-Coyle et al., 1992; Wheeler et al., 1993).

The adaptive value of circadian clocks for fitness has been demonstrated by diverse strategies that have been nicely reviewed by Vaze and Sharma (2013), Abhilash and Sharma (2016), and Nikhil and Sharma (2017). One of many possible strategies is to investigate whether a trait confers higher adaptive advantage in the context of species competition. For example, Ouyang et al. (1998) and Dodd et al. (2005) showed that organisms with an endogenous period (τ) close to the period of the Zeitgeber (T) have a greater competitive advantage over those with a deviant τ. Ouyang et al. (1998) have grown a wildtype cyanobacteria strain in competition with mutants possessing too fast, too slow or no clock at all for about 50 generations. In this competition assay, the mutants lost against the wildtype strains when grown under a 24-h day. However, when grown under environmental period lengths (“T-cycles”) that matched the endogenous circadian period of the mutants, the mutants outcompeted the wildtype strain, respectively. The result shows that cyanobacteria have a significant competitive advantage under T-cycles that resonate with their endogenous free-running period, because under such conditions they achieve an optimal phase relationship between the light-dark (LD) cycle and the endogenous clock - also known as the resonance hypothesis (Pittendrigh and Minis, 1972; Ouyang et al., 1998; Johnson et al., 2008). Dodd et al. (2005) performed a similar experiment in the plant, *Arabidopsis thaliana*, during one vegetative growth season. Mutant and wildtype plants were grown in competitions together under different T-cycles and – similar to cyanobacteria – the plants whose endogenous periods matched with the external T-cycle had more photosynthesis and growth and enhanced survival.

In mammals, researchers tried to demonstrate the importance of the circadian clock for fitness under natural conditions, under which the animals are exposed to harsh weather conditions, competition for food and the risk of predation. DeCoursey and colleagues compared the survival of chipmunks with surgical ablated circadian clock in the suprachiasmatic nucleus of the hypothalamus with that of sham-operated siblings for 18 months under natural conditions in a high-density population of free-living eastern chipmunks at a 4-ha forest site in the Allegheny Mountains (United States) (DeCoursey and Krulas, 1998; DeCoursey et al., 2000). They found that a significantly higher proportion of clock-ablated animals than sham-operated individual were killed by weasel predation, most probably because the clock ablated animals showed nocturnal restlessness and left their burrows more frequently at night when predator risk is highest for these diurnal animals. DeCoursey (2014) replicated these experiments with further rodent species, nocturnal flying squirrels, diurnal Antelope squirrels and diurnal Golden-mantled ground squirrels. In all species the circadian clock significantly reduced the time being awake during the daily sleep times (for Golden-mantled ground squirrel also during hibernation), and by this way helped to save energy and to avoid predation.

Nevertheless, other studies did not reveal any benefits of possessing a circadian clock. For example, clock mutant (*per2^*Brdm*1^*) mice that were kept for 2 years in 400 m^2^ outdoor pens within a remote woodland in Western Russia showed survival rates that were equal to wild-type mice (Daan et al., 2011). Although the circadian organization and entrainment of *per2^*Brdm*1^* mutants is compromised in the laboratory (Albrecht et al., 2001; Mendoza et al., 2010), their activity pattern did not differ from that of wild-type mice in nature (Daan et al., 2011). This result questions the importance for a functional clock under natural conditions. Vanin et al. (2012) came to similar conclusions with regard to fruit flies that were kept over the summer under semi-natural conditions: clock-less *per*^0^ mutants exhibited similar bimodal activity patterns with prominent morning and evening activity bouts as did wild-type flies, and there was only a marginal difference in the timing of evening activity between wild-type flies and *per*^*s*^ and *per*^*l*^ mutants. Similar results were obtained in the laboratory when either realistic temperature and light cycles were simulated (Vanin et al., 2012) or when only realistic light cycles were simulated (Schlichting et al., 2015). This suggests that more naturally cycling environmental stimuli than the standard ones in the laboratory allow even mutant flies to behave normally. Schlichting et al. (2015) found that the eyes play an essential role in this process. Eyeless *per*^0^ mutants lacked the normal bimodal organization of activity, suggesting that functional eyes can largely compensate for the loss of the circadian clock by directly modulating the activity of the flies, which is also known as masking effect. Although in fruit flies no survival tests were performed under outdoor conditions and in mice these lasted only for 2 years and might be too short to draw strong conclusions, the natural-like activity patterns of arrhythmic mice and flies under natural conditions due to masking may explain, why none or only a minor impact on the mutants’ fitness was found.

**FIGURE 1:**
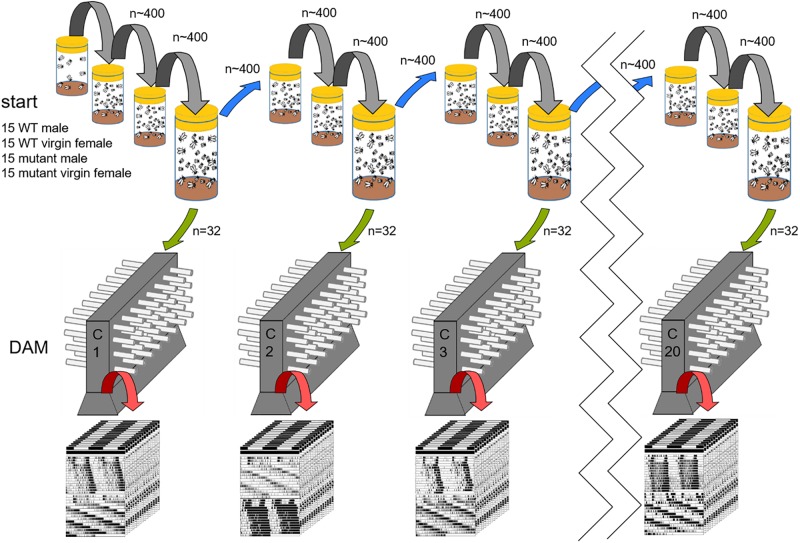
Experimental procedure. At the start of the experiment, 15 male and virgin female flies each of wildtype (WT) and one *period* mutant were placed in a large food vial (60 flies in total). The mutant flies were either *per*^*s*^, *per*^*l*^ or *per*^0^. All wild type/mutant competitions were started with 10 vials (=populations) each. Here, the procedure is only shown for one population. Every 14 days (gray and blue transfer arrows), all hatched animals (∼ 400 flies) were transferred into fresh food vials. After every third transfer, a census was taken. For this, the vials were kept for another 2 to 3 days so that more flies could hatch. From these, 32 males were randomly chosen (green arrow) and their activity was recorded in a *Drosophila* Activity Monitor (DAM), [Census 1 (C1), Census 2 (C2), etc.]. After 4–7 days of entrainment under light dark cycles (LD12:12) the animals were recorded in constant darkness (DD) for another 2 to 3 weeks. Afterward the genotype of the flies was determined and the period of the rhythmic flies calculated (see section “Materials and Methods”).

In the laboratory, when fruit flies live under comfortable, constant temperatures, are provided with super-abundant food, and lack any competition with flies of other genotypes as well as any predation risk, a properly running circadian clock may not be essential for survival, although the activity patterns of wildtype flies and clock mutants are quite different under these conditions (Figure 1). Klarsfeld and Rouyer (1998) and Vaccaro et al. (2016) observed only tiny differences in lifespan between *per*^0^, *per*^*T*^ (another short-period mutant) and *per*^*l*^ and wildtype flies. Wildtype flies lived marginally longer than the mutants under a 24-h day, while under a 16-h day, that mimics the period of *per*^*T*^ mutants, the latter did not live longer than wild-type flies and even no longer than *per*^*l*^ mutants (Klarsfeld and Rouyer, 1998). The results suggest that the influence of the clock on lifespan is rather small and does not follow the resonance hypothesis.

Nevertheless, most of the mentioned studies have certain limitations (see Abhilash and Sharma, 2016). Some focused on a single readout of fitness (e.g., lifespan) and did not regard others (e.g., fecundity) that may have compensatory effects on overall fitness (see Sheeba et al., 2000 for an example). Other studies used mutants that are inbred in the lab for many years and might have undergone random genetic drift producing spurious correlations between different fitness traits. Thus, it is premature to conclude from these studies that the circadian clock plays only a minor role in fitness, even under such “comfortable” lab conditions.

Here we report a long-term study, in which we raised the original *period* mutants, *per*^0^, *per*^*s*^, and *per*^*l*^ (Konopka and Benzer, 1971), in competition with wildtype flies for more than 2 years (>50 generations) and under different T-cycles, while regularly determining the proportion of mutant flies in the population. Such a long-term study was, so far, only performed on the already mentioned cyanobacteria that have a very short generation time (Ouyang et al., 1998), but not in any animal. Although fruit flies have a significant longer generation time than cyanobacteria (∼14 days in comparison to ∼10 h), it appeared feasible to us to perform this experiment with fruit flies. In addition, we performed a short-term competition study (over three generations in two consecutive years) under outdoor conditions to test the impact of the circadian clock on fitness under more natural-like climatic and light conditions. Both, the long-term and short-term competition study, showed that a properly running circadian clock significantly improves the fitness of fruit flies under competition. Furthermore, the long-term competition study did partly obey the resonance hypothesis. Most interestingly, in spite of losing against wild-type flies under a 24-h day, the *per*^*s*^ and *per*^*l*^ mutants stably remained at low percentages in the fly population. To understand this, we additionally performed fertility, survival and mating preference tests.

## Materials and Methods

### Fly Strains and Rearing

Wildtype CantonS flies (WT_CS_) and the *period* mutants (*per*^*s*^, *per*^*l*^, and *per*^01^) from Konopka and Benzer (1971) were used for the experiments. In the following the *per*^01^ mutants will just be named *per*^0^. We obtained the flies from the Kyriacou group, who state in their paper that the flies are congenic and cantonized (Vanin et al., 2012). We could not obtain further information about the number of performed crosses with CantonS flies, but the flies have been co-isogenised for 12 generations in 1992 as is described in detail in Greenacre et al. (1993). Flies were reared on cornmeal/agar medium consisting of 0.8% agar, 2.2% sugar-beet syrup, 8.0% malt extract, 1.8% yeast, 1.0% soy flour, 8.0% corn flour, and 0.3% hydroxybenzoic acid at 25°C and 60% relative humidity under a light-dark (LD) cycle of 12:12 h (h). We started the first competition experiments under *T* = 24 h in 2013 (running until 2015) and the second ones, depending on the mutant either under long and short T-cycles or in LL in 2014 (running until 2016).

### Long-Term Competition Assay in the Laboratory

Ten food vials (diameter 48 mm, height 104 mm, filled with 28.5 ml food), each containing 15 virgin female and 15 male flies each of wild-type (CS) and *period* mutant flies were used to start the competition experiments (in sum, 60 flies per vial). The mutant flies were either *per*^*s*^, *per*^*l*^ or *per*^0^ (Table 1). All vials were kept in a climate chamber at 25°C ± 0.2°C with 60% ± 2% of relative humidity. The light condition was either a T-cycle of 24 h (LD 12:12), a T-cycle of 29 h (LD 14.5:14.5) or of 19 h (LD 9.5:9.5) or constant light (LL) depending on the *period* mutant flies used for the subset of the experiment (Table 1). Light intensity was always 100 lux. LL conditions were only used for competing wildtype flies and *per*^0^ mutants, because wildtype flies become arrhythmic under LL. Thus, the wildtype activity pattern is no longer distinguishable from that of the arrhythmic *per*^0^ mutants and any selective advantages of possessing a circadian clock should disappear.

**TABLE 1:** Experimental settings of long-term competition experiments.

	**Condition 1**	**Condition 2**
WT_CS_ × *per*^*l*^	LD 12:12	LD 14.5:14.5
WT_CS_ × *per*^*s*^	LD 12:12	LD 9.5:9.5
WT_CS_ × *per*^0^	LD 12:12	LL

*In each case condition 1 mimics the standard 24 h cycle whereas condition 2 that (approximately) resonating the endogenous rhythm of the mutant involved in the experiment.*

At 25°C, the generation time of fruit flies is ∼10 days and we did not observe any evident differences in developmental timing in the mutants. Therefore, we transferred all flies from the vials to new vials with fresh food every 14 days. At this time, ∼400 flies of the new generation had eclosed. The parental flies had died before the flies were flipped (when the larvae grow, the food becomes soft and fluid and the adults submerge in it being finally eaten by the larvae). Every third generation, the old vials were kept for another 2 to 3 days for more flies to hatch to carry out a census on mutant allele frequency (Figure 1). At these censuses, 32 males from each vial of these later hatching flies where used for locomotor activity recording to determine the genotype of each fly. Since the *period* gene is on the X-chromosome of which males carry only one copy, the genotype of each male becomes evident in its free-running period (short, long, ∼24 h or arrhythmic). Based on these tests, the genotype distribution among all flies in the experimental vials was determined (see below). In sum, 320 males (stemming from 10 vials) were used for determination of male genotype distribution at every census. This procedure was repeated over 60 generations for the 24 h T-cycle experiment and over 54 generations for the short- and long T-cycles. Figure 1 illustrates the procedure. Most importantly, the population size in the single vials stayed approximately constant throughout the entire experiments as far as we could judge by eye; this observation suggests the existence of a carry capacity and thus of competition among flies, respectively, larvae.

### Outdoor Competition Assay

**FIGURE 2:**
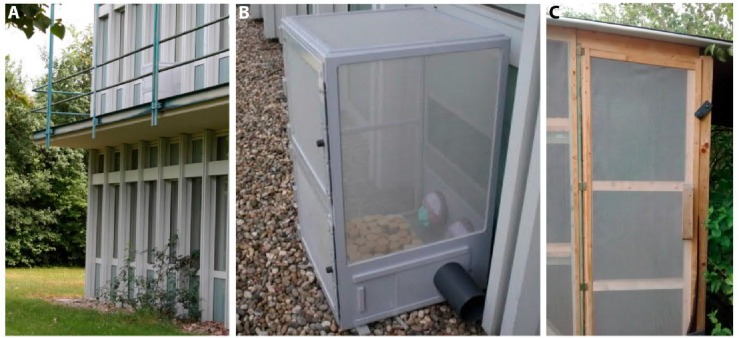
Location of the vials during the outdoor experiment in the summer months. **(A)** and **(B)** show the location at the Biocenter, 1st floor, **(C)** the sheltered location at the Bee station of the University of Würzburg.

The outdoor experiments were performed in 2013 and 2014 outside the Biocenter and at the bee-station of the University of Würzburg – sheltered from rain and direct sunlight – where the flies could sense all changes in light, temperature and relative humidity (Figure 2). The outline of the competitions assay was in principle similar to that described above. However, due to low and variable temperatures, the flies took far more time to develop and to generate the next generation. Therefore, only three generations could be raised during each summer period (June to September/early October). The third generation was investigated for the genotype distribution of WT_CS_ and *period* mutant flies as described above. We compared these data with the third generation data (=census 1) from the long-term indoor experiments, using a general linear model with a “quasibinomial” error structure due to overdispersion of data. As suggested by Crawley (2013) we used *F*-tests for model comparison (procedure “anova” in R) – separately for each mutant – between the full model and a model version with the effect of “year” removed (the 2 years of outdoor experiments and 1 year of indoor experiment). Even though these constitute three independent experiments we apply *post hoc* Bonferroni adjustment for multiple testing.

### Locomotor Activity Recording and Analysis

Locomotor activity was recorded in *Drosophila* Activity Monitors (DAM; Trikinetics system, Waltham, MA, United States) at 25°C and 100 lux as described previously (Schlichting and Helfrich-Förster, 2015). Light was provided by white LEDs (Lumitronix, LED Technik, Hechingen, Germany). We recorded the activity of male wildtype, *per*^*s*^, *per*^*l*^ and *per*^0^ flies (1) before the competition experiments, (2) at the censuses during the competition experiments and (3) in parallel to the competition experiments, in flies of each genotype that were kept without competition under the same conditions. The flies were recorded for 4–7 days under LD 12:12 cycles (light intensity 100 lux) and consecutively for 2–3 weeks under constant darkness (DD). In addition, we recorded male wildtype flies and *per*^0^ mutants under constant light of 100 lux to confirm that the great majority of flies in both strains are arrhythmic under such conditions. Furthermore, we recorded the activity of male wildtype flies and *per*^*s*^ and *per*^*l*^ mutants under the different T-cycles (*T* = 19 h and *T* = 29 h) in order to see how the phasing of their activity bouts changed under these conditions.

The raw activity data was exported as text files by the DAM-System Software, displayed as actograms using a Fiji^1^ plugin – ActogramJ (v0.9, (Schmid et al., 2011) and saved as pdf files. Average activity profiles during the LD-cycles were calculated for each genotype as described in Schlichting and Helfrich-Förster (2015). From the average activity profile the fly’s overall activity (=number of beam crosses) during the first 3 h of the day [Zeitgeber Time (ZT) 0 to 3] was determined for each individual fly. Data were tested for normal distribution and investigated for genotype influences by a One-Way ANOVA followed by a *post hoc* test with Bonferroni adaptation. To determine rhythmicity and the endogenous free-running period of every single fly in DD, the raw data were analyzed by χ^2^-periodogram analysis in ActogramJ. At *p* > 0.05 the flies were assumed to be arrhythmic. From the periodogram analyses, the proportion of wildtype and mutant flies was determined for each competition pair in the competition assay, and the frequency to which the mutant flies persisted in the population was plotted for every census in a diagram.

### Diverse Assays to Determine Fitness Components of the Clock Mutants

Overall fitness depends on several components, e.g., fertility, mating success, survival during development, number of offspring, etc. In order to reveal the fitness of the clock mutant in comparison to wildtype flies in more detail, we determined several fitness components. All tests were done under LD12:12 with 100 lux during the light period and a constant temperature of 25°C.

#### Sperm Counts

Pairs of flies of the same genotype were placed in mating chambers and allowed to mate. After successful copulation, the female reproductive organs – consisting of the uterus, seminal receptacle and the spermatheca were dissected in PBS, transferred to a glass slide and stained with DAPI (1 μg/ml in PBS). The preparations were scanned with a Leica confocal laser scanning microscope (DM5500; stack width 2 μm). The sperms heads in the seminal receptacle, the uterus and the spermathecal were counted with ImageJ (Fiji) via defining ROIs and automated counting as described in Garbaczewska et al. (2013). Mating and sperm counts were taken for 8 wildtype flies, *per*^0^ and *per*^*l*^ mutants, respectively, and 7 *per*^*s*^ mutants. Sperm count data were first tested for conforming with normality assumption using the Shapiro test and for homogeneity of variance using the Bartlett test; both tests provided no evidence of significant violations of these assumptions. As we were interested in the comparison between the wildtype *CS* and the three mutants only, we then carried out *post hoc* tests with Dunnett correction (utilizing the “emmean” package, Lenth, 2019, in R) only contrasting each mutant to the CS wildtype.

#### Survival From Egg to Adults

To test whether survival rates during development are different between wildtype and mutant flies, females of each genotype were allowed to lay eggs on apple agar plates for 6 h. From these, 100 eggs, were transferred into 10 food vials, respectively, and the pupae and adult flies emerging from these eggs were determined. The egg-pupae-adult survival data were analyzed using a generalized model with a “quasibinomial” error family for the egg to pupae survival due to overdispersed data and the standard “binomial” family for the survival from pupae to adults. Significance testing was thus based on the *F*-ratio test for the former but on the likelihood-ratio test with *Chi*^2^ statistics for the latter type of analysis (Crawley, 2013). For the egg to pupae survival all experiments started with exactly 100 eggs per vial that had been laid by a group of females within few hours. For each genotype we had 10 such vials (=1000 eggs in total for each genotype). The number of pupae produced provided, in turn, the initial sample for then testing the survival from the pupal to the adult stage. Again, we were primarily interested in comparing the different mutant to the wildtype and thus performed pairwise testing of mutant homozygotes versus the mutant wildtype females. A similar approach (Dunnett’s correction) as for the sperm count data was used for *post hoc* testing, contrasting only the mutants with the wild type.

#### Female Mating Success and Preferences

**FIGURE 3:**
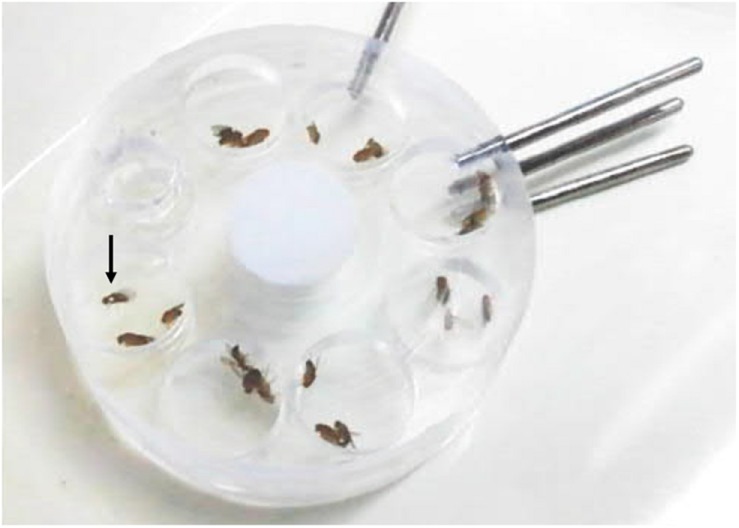
Mating wheel with seven mating chambers. Each chamber is filled with one female and two male flies, respectively, for testing female mating preferences. One of the two males is marked by a white spot on its thorax (arrow) to distinguish the genotypes.

Individual single female flies – heterozygous or homozygous for either WT_CS_ or *period* mutant – and two male flies (a WT_CS_ and a mutant) were placed in mating chambers (Figure 3). The males were allowed to court for 2 h. If females did not mate within this time interval, the flies were discarded and new flies were tested. Overall, 280 experiments were conducted with heterozygous and 140 experiments with homozygous females of each type. If one male managed to copulate successfully with the female, its genotype was noted and it was left undisturbed until copulation ended (usually this lasted ∼25 min). To distinguish between the two males with different genotypes, one of the two males was marked by a white spot on the thorax between the wings. The experiments were conducted at ZT 0–2 during the flies’ morning activity.

The overall mating success was determined and analyzed using a general linear model with “quasibinomial” error structure, including the genotypes of the mutant males (*per*^*s*^, *per*^*l*^ or *per*^0^), of the females (WT/WT, WT/mutant, and mutant/mutant). Model comparison was carried out by backward elimination using *F*-ratio tests as described before.

The analysis of mating preferences was complicated because one of the two males presented to the females had to be marked to distinguish the mutant from the wildtype male (mutants do not carry external markers). In a given setting it is thus not clear whether a female took her choice based on the genotype of the male or the presence/absence of the marker. Experiments were thus replicated in two versions, in one the wildtype males carried the mark in the others the mutant male. In our analysis we thus need to separate the effect of being a mutant (*a*_*m*_) from that of being marked (*a*_*x*_) on the underlying “base attractiveness” (*A*) of males (note that we assume additive effects only). Consequently, the two setting provide the following ratios for each female type tested (for clarity we drop index *i* indicating the different female genotypes in the following):

(1) $$\alpha =\frac{A+a_{m}}{A+a_{x}}\;\text{or}\;\rho_{\alpha }=\frac{A+a_{m}}{2\,A+a_{x}+a_{m}},$$

with *p*_α_ the probability of choosing an unmarked mutant over a marked wild-type male and

(2) $$\frac{1}{\beta }=\frac{A+a_{m}+a_{x}}{A}\;\text{or}\;\rho_{\beta }=\frac{A+a_{m}+a_{x}}{2\,A+a_{x}+a_{m}},$$

with *p*_β_ the probability of choosing a marked mutant over an unmarked wild-type male. These are two equations with three unknown variables. However, we are only interested in the relative attractiveness of mutant vs. wild-type males, i.e.,

(3) $$\frac{A+a_{m}}{A}=v_{m},$$

or alternatively the probability ρ_*m*_ that a female of genotype *i* will choose a mutant male over the wild-type, i.e.,

(4) $$\rho_{m}=\frac{A+a_{m}}{A+A+a_{m}}={\left(\frac{A}{A+a_{m}}+1\right)}^{-1}={\left(1+\frac{1}{v_{m}}\right)}^{-1}=\frac{v_{m}}{1+v_{m}}.$$

Simple algebraic manipulations yields the following equation

(5) $$v_{m}=\alpha \,\left(1+\frac{1-\beta \,\alpha }{\beta \,\left(1+\alpha \right)}\right)$$

that allows calculating the desired values for *p*_*m*_ (see Eq. 4).

Calculation of confidence intervals for *p*_*m*_ is complicated by this fact, however, and not liable to standard methods. We thus implement a Monte-Carlo approach to estimate confidence intervals for values of *p*_*m*_ by exactly replicating the numerical calculations of Eqs 4 and 5 but drawing random values for the number of mutant males selected by females from a binomial distribution with probabilities for *p*_α_ and *p*_β_ as empirically estimated from the experiments (cf. Eqs 1 and 2) and sample sizes exactly as those in the experiments. This random drawing of $p_{\alpha }^{r}$ and $p_{\beta }^{r}$ and calculation of $p_{m}^{r}$ is replicated 100,000 times for each of the female genotypes x male genotype combinations. The 2.5 and 97.5% quantiles of the distribution of these $p_{m}^{r}$ values are then used to specify the 95% confidence limits of *p*_*m*_. We are primarily interested in the presence of a non-random mating preference, i.e., whether the preference is significantly different from *p*_*m*_ = 0.5. A significant mating preference is thus indicated if the 95% CI-Interval does not include the value of 0.5.

#### Offspring per Female Fly

The mated females from the mating preference tests were used to determine the number of offspring per female fly. Each female was allowed to lay eggs for 3 days into one food vial. Then the female was removed and the emerged offspring was counted after 14 days at 25°C. Due to the discrete nature of the offspring count-data, they were analyzed using a generalized linear model with a “quasipoisson” error structure and a *post hoc* testing with Bonferroni correction. Again, statistical significance testing and model simplification was based on model comparison using the *F*-ratio test.

### Statistics

All statistics were performed with R, version 3.4.4 (R Core Team, 2018).

## Results

### Activity Profiles of Wildtype and Mutant Flies Under 12:12 LD Cycles, Constant Darkness, Constant Light and 19 h/29 h T-Cycles

**FIGURE 4:**
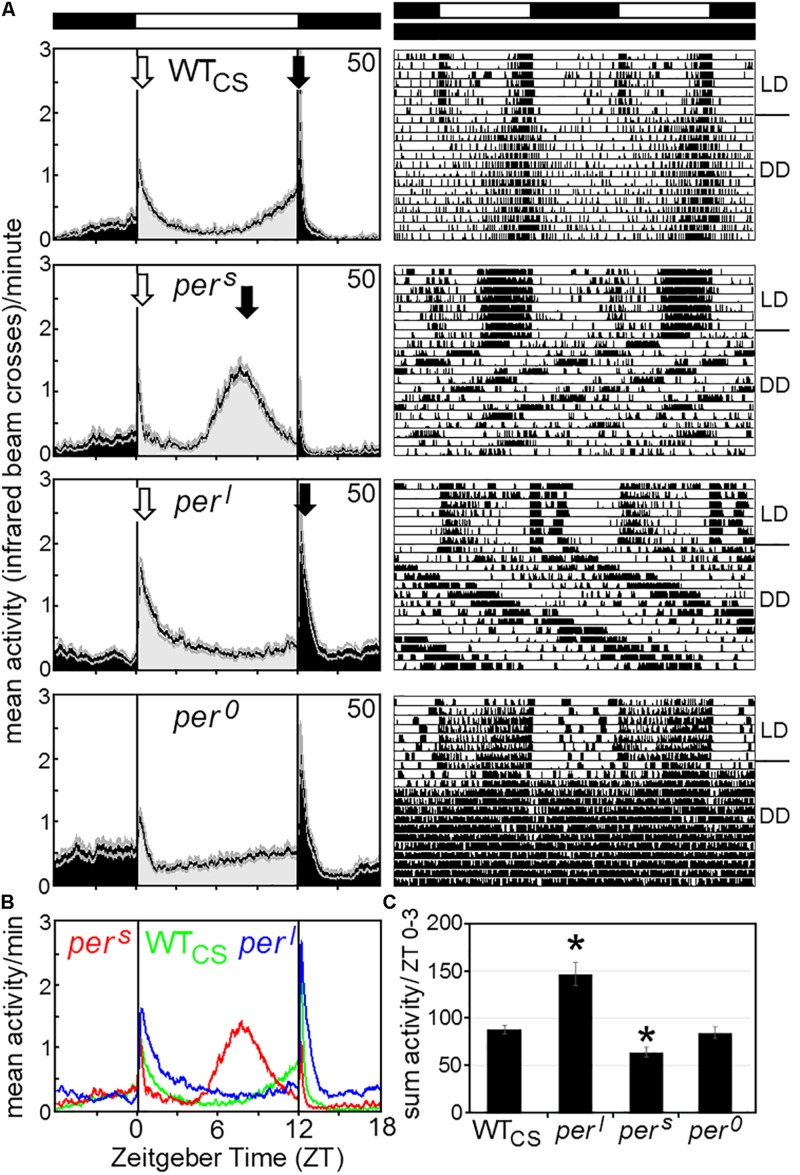
Average activity profiles and typical actograms of individual wildtype (WT_CS_) and *period* mutant (*per*^*s*^, *per*^*l*^ and *per*^0^) flies under LD and constant darkness (DD). **(A)**: Under LD cycles, WT_CS_ flies show a bimodal activity pattern with morning (open arrow) and evening (closed arrow) activity bouts. In *per*^*s*^ mutants the evening activity bout is advanced into the early afternoon while it is delayed into the night in *per*^*l*^ mutants. The morning activity bout appears to be reduced in *per*^*s*^ mutants, while it is pronounced and long-lasting in *per*^*l*^ mutants [see also **(B,C)**]. *per*^0^ mutants are active during the day and night and strongly respond to lights-on and lights-off, but lack evident morning and evening activity bouts. Under DD, the actograms (right diagrams) show that the WT_CS_ fly free-runs with an endogenous period of ∼24 h, while the *per*^*s*^ mutant free-runs with a short period, the *per*^*l*^ mutant with a long period and the *per*^0^ mutant becomes arrhythmic. **(B)**: Overlay of the average activity profiles of *per*^*s*^, WT_CS_ and *per*^*l*^ flies, showing the different phases of the evening activity bout and the different activity levels during morning activity. **(C)**: Sum of activity (beam crosses) during the first 3 h (ZT0-3) after lights-on for all fly strains. Significant differences to WT_CS_ flies are indicated by asterisks.

**FIGURE 5:**
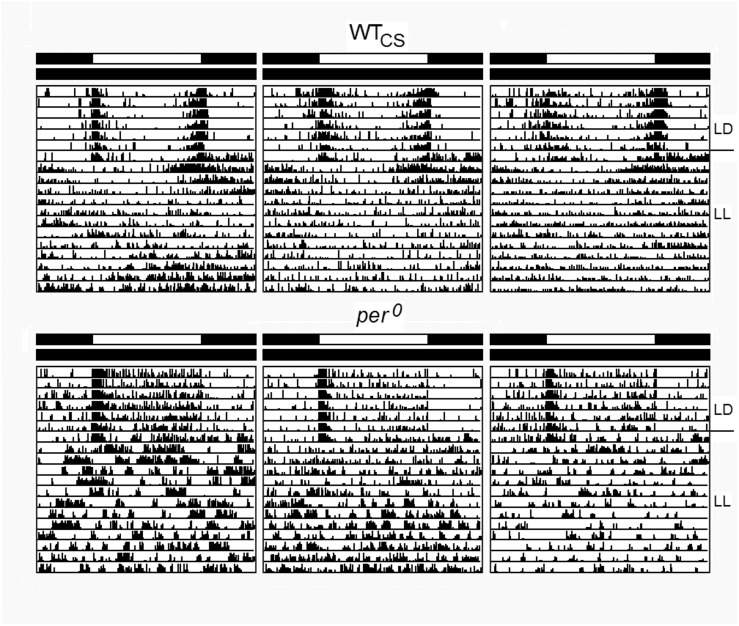
Typical actograms of wildtype flies (WT_CS_) and *per*^0^ mutants under LD and constant light (LL). For both strains three individual actograms are selected that revealed the strongest rhythmicity under LL. Of 32 recorded wildtype flies 6 revealed weak rhythms with a mean period of 26.0 ± 0.6 h (SEM). The other flies were arrhythmic. In *per*^0^ mutants, periodogram analysis found weak rhythms in 5 of 32 flies. These had a mean period of 25.0 ± 1.4 h (SEM). All other flies were arrhythmic.

Our first aim was to compare the activity patterns of wildtype flies and the *period* mutants under LD 12:12 and constant darkness (DD) with the reported data and to check whether *per*^0^ and wildtype flies behave similarly arrhythmic under constant light (LL). Figure 4A shows typical actograms and activity profiles for wildtype flies, *per*^*s*^, *per*^*l*^, and *per*^0^ mutants under LD and DD, while Figure 5 depicts typical actograms of wildtype flies and *per*^0^ mutants under LD and LL. Under LL, the great majority of wildtype and *per*^0^ flies were arrhythmic (details see legend of Figure 5). Under DD conditions, only *per*^0^ mutants exhibited arrhythmic activity patterns, all other fly strains were clearly rhythmic, whereby wildtype flies free-ran with a period close to 24 h, *per*^*s*^ with short period and *per*^*l*^ with long period (Figure 4; detailed period calculation see in section “Changes in free-running period over the course of the indoor competition experiment”). As reported previously (Hamblen-Coyle et al., 1992), wildtype flies exhibited two activity bouts around lights-on and lights-off (also called morning and evening peaks). Morning and evening activity anticipated lights-on and lights-off, respectively, and were separated by a siesta. *per*^*s*^ mutants showed a much earlier and shorter siesta and their evening activity occurred already in the early afternoon. The morning activity might take place already before lights-on, but it seemed largely suppressed by darkness. In contrast, *per*^*l*^ mutants, exhibited a large morning activity that extended until midday. The following siesta was very late and stretched until lights-off. Evening activity started after lights-off and appeared largely suppressed by darkness, as was the case for the morning activity of *per*^*s*^ mutants. The calculation of the activity amounts during the first 3 h of the day, revealed significant differences between wildtype flies and *per*^*s*^ and *per*^*l*^ mutants (Figure 4C): *per*^*l*^ mutants were much more active than wildtype flies, while *per*^*s*^ mutants were less active. *per*^0^ mutants lacked the siesta and showed no evident morning and evening activity bouts, but instead merely responded to lights-on and lights-off.

**FIGURE 6:**
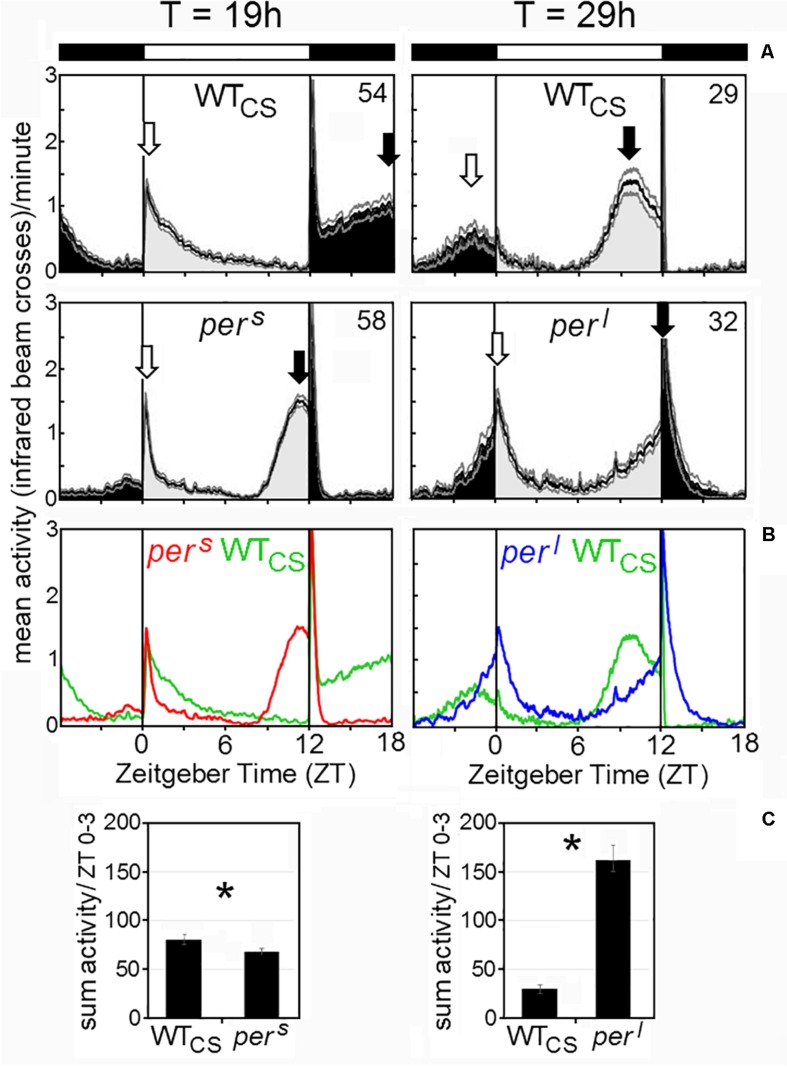
Average activity profiles of wildtype (WT_CS_) and *period* mutant (*per*^*s*^, *per*^*l*^) flies under T-cycles of 19 and 29 h. **(A)**: The flies show a bimodal activity pattern with morning (open arrow) and evening (closed arrow) activity bouts. In wildtype flies, morning and evening activity are delayed under 19 h T-cycles, while they are advanced under 29 h T-cycles. In *per*^*s*^ and *per*^*l*^ mutants the timing of morning and evening activity under 19 h T-cycles and 29 h T-cycles, respectively, resembles the timing in wildtype flies under 24 h cycles (compare Figure 1). **(B)**: Overlay of the average activity profiles of *per*^*s*^, WT_CS_ and *per*^*l*^ flies, showing the different phases of the evening activity bout and the different activity levels during morning activity. **(C)**: Sum of activity (beam crosses) during the first 3 h (ZT0-3) after lights-on for all fly strains. Significant differences to WT_CS_ flies are indicated by asterisks.

Under long and short T-cycles, the activity profiles of wildtype flies and *per*^*s*^ and *per*^*l*^ mutants changed drastically (Figures 6A,B). Under short T-cycles (19 h), wildtype flies did not at all anticipate lights-on but showed instead a long-lasting pronounced morning activity resembling very much the morning activity of *per*^*l*^ mutants under 24-h cycles (Figure 4A). Evening activity of wildtype flies shifted completely into the night, even more than that of *per*^*l*^ mutants under 24-h cycles. Under long T-cycles (29 h), wildtype flies showed early morning and evening activity, again resembling the activity profiles of *per*^*s*^ mutants under 24 h cycles with the exception that the nocturnal morning activity of wildtype flies was clearly visible, while it appeared suppressed in *per*^*s*^ mutants. The mutants’ activity profile under 19 and 29 h cycles, respectively, came close to the activity profile of wildtype flies under 24-h cycles. Overall, this experiment revealed that, under the T-cycles that matched the endogenous period of the mutants, the mutants had an almost “wildtype-like” phase-relationship of morning and evening activity to lights-on and lights-off, while wildtype flies behaved “mutant-like”. Consequently, the mutants should have a selective advantage over the wildtype flies under T-cycles, at least if fitness depends solely on the phasing of their activity bouts.

### Indoor Competition Experiments

**FIGURE 7:**
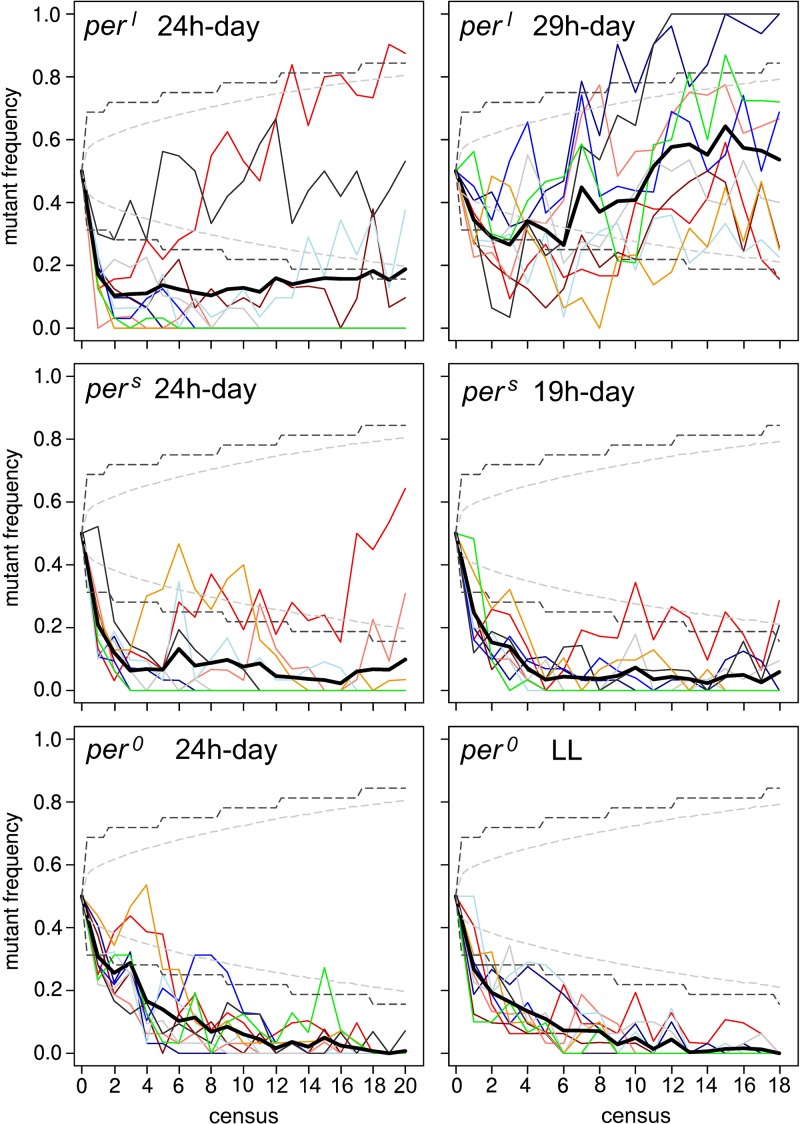
Results of the competition assay experiment in 24-h, 29-h and 19-h days (for *per*^*l*^ and *per*^*s*^ mutants, respectively) and 24-h and constant light (LL) for *per*^0^ and WT_CS_. For each assay 10 food vials with the *period* mutants competing with wildtype flies were established. The mutant frequency per generation was determined separately in each vial (thin colored lines) and averaged over all vials (thick black line). Hatched gray lines show simulated 95% confidence limits for the frequency in the whole population in scenarios assuming pure genetic drift (identical fitness of wild-type and mutant) and hatched black lines the corresponding 95% confidence limits for samples of 32 males as taken in the experiments (see text for more details on the creation of these intervals; for details see section “Materials and Methods”). Except for the assay in which *per*^*l*^ mutants competed with wildtype flies under the 29 h-day (top right), on average the wildtype flies dominated the *period* mutant flies in all experiments. Nevertheless, in some vials the *per*^*l*^ and *per*^*s*^ mutants performed better than the wildtype flies even in the 24 h-day, so that the mutants persisted in the overall population.

Figure 7 depicts the sampled frequency at which the *period* mutant flies persisted in the fly population over time when grown in competition to WT_CS_ flies. We directly compared the outcome of the experiment under the 24-h cycle with the outcome under the 29-h T-cycle for the *per*^*l*^ mutants, with the outcome under the 19-h T-cycle for the *per*^*s*^ mutants and with the outcome under LL for the *per*^0^ mutants, respectively. Under the 24-h day, the frequency of the mutants quickly declined in most of the food vials for all three *period* mutants (Figure 7 left). While *per*^0^ mutants declined to low proportions in all food vials (both, 24-h cycle and LL) and presumably completely disappeared from several vials (what we cannot say with certainty, as we only genotyped a fraction of the male population on each census), the *per*^*l*^ mutants persisted in at least 4 vials (in one of them to 80% and in another one to 40%) under 24-h cycle and in all ten vials under the 29-h cycle. *per*^*s*^ mutants persisted in at least 3 vials under both T-cycles. On average, *per*^*l*^ and *per*^*s*^ mutants remained to a certain low percentage in the population (*per*^*l*^ ∼20%, *per*^*s*^ ∼5%; Thick line in Figure 7 left).

To test whether these results could also be explained by genetic drift, we created 95% confidence intervals for the “true” frequency of mutant males in the population and (hatched gray lines in Figure 7) and just for samples of 32 males (hatched black lines) under the assumption of pure genetic drift. Because only males were genotyped, the *period* mutants are linked to the sex chromosome, data sets represent time-series, and a census was only taken every 3rd generation, a direct (analytical) calculation of confidence intervals is not possible in this case. Instead, we simulated the competition experiments exactly replicating the protocol of population initialization, generation transfer, and random sampling of 32 males every 3rd generation as applied in the experiments but under the (neutral) assumption of identical fitness of wildtype and mutant genotypes and the assumption of random mating and random sex determination. To account for sampling effects at any of these steps, discrete values for the number of females and males as well as the different genotypes by drawing random values from a binomial (two sexes, two possible male genotypes) or multinomial (three female genotypes) distribution, were assumed. These simulations were replicated 100,000 times and the lower and upper 2.5% quantiles were then used to define the 95% confidence intervals for both, the frequency of the mutant allele in the whole population and for the frequency in just a sample of 32 males. The calculated confidence intervals are shown in Figure 7. The results for the *per*^0^ and the *per*^*s*^ mutants can clearly not be explained by genetic drift alone but indicate strong selection against the mutants in both, the 24-h cycles and the LL, respectively, the 19-h cycle. For *per*^*l*^, however, the results under the 29-h cycle are consistent with genetic drift and also under the 24-h cycle some of the populations show fluctuations in the mutant’s frequency consistent with genetic drift (but see below for deviations from the genetic drift hypothesis).

If the decrease of the *period* mutants in the fly population depends only on the presence and speed of circadian clock, *per*^*l*^ mutants are expected to improve performance against wildtype flies under the 29-h T-cycle and *per*^*s*^ mutants under the 19-h T-cycle, while *per*^0^ mutants should be equally fit as wildtype flies under LL conditions. However, only *per*^*l*^ mutants performed better under the 29-h T-cycle than under the 24-h cycle, while *per*^*s*^ and *per*^0^ mutants performed equally poor under the 19-h T-cycle and LL as they did under the 24-h cycle, respectively (Figure 7 right). Under the 29-h T-cycle, *per*^*l*^ mutants persisted in all 10 vials to varying percentages, on average to ∼50%. In two vials, the mutants outcompeted the wildtype flies, meaning that in the end they represented presumably 100% of the population. This cannot be due to genetic drift. The final frequency of *per*^*l*^ mutants in the other vials are within the confidence intervals of genetic drift and might theoretically be caused by it (see above). However, the strong decline of the mutant frequency at the beginning of the experiment (lasting until census 6) that is followed by a clear increase in frequency are hard to reconcile with genetic drift. In particular, the comparison between the shape of the “frequency curves” at *T* = 24 h and *T* = 29 h shows that *per*^*l*^ mutants perform clearly better under the 29-h day, which supports the resonance hypothesis. Nevertheless, the poor performance of the *per*^*s*^ and *per*^0^ mutants under both T-cycles clearly shows that additional factors contribute to the fitness of the mutants. These factors can be related or completely independent of the circadian clock.

### Outdoor Competition Experiments

**FIGURE 8:**
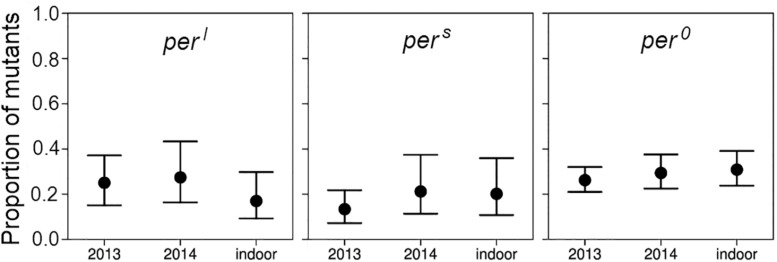
Proportion and binomial 95% CI-intervals of mutant males after three generations of competition with wildtype flies under outdoor and indoor conditions. Data stem from the experiments carried out under external (natural) light conditions in 2013 and 2014 and from the third generation census of the long-term competition experiments under the 24-h day in the laboratory (see Figure 7).

In the outdoor experiments, the generation time of the flies was ∼1 month and we could breed through only three generations in each summer. In both summers, 2013 and 2014, the wildtype flies clearly outnumbered the mutants in the third generation (Figure 8). Roughly speaking, the mean proportion of the mutant fell from 0.5 to about 0.25 in all the experiments significantly deviating from the initial 1:1 ratio (*p* < 0.001) indicating selection against the mutant alleles. There was no significant difference in the percentage of remaining mutants between the 2 years under outdoor conditions and also not between the outdoor and indoor conditions (d.f. 2, 23, deviance reduction = 10.25, *F* = 3.065, *p* > 0.05 following *post hoc* Bonferroni correction). The fact that the decline in the mutant abundance was about similar in the out- and indoor experiments suggests that the different conditions, in particular also the differences in the light regime, between indoor and outdoor conditions do not have a relevant impact on the mutants’ success. However, different selective response may also not have been possible due to limited genetic variance in the inbred initial populations.

### Changes in Free-Running Period Over the Course of the Indoor Competition Experiment

**FIGURE 9:**
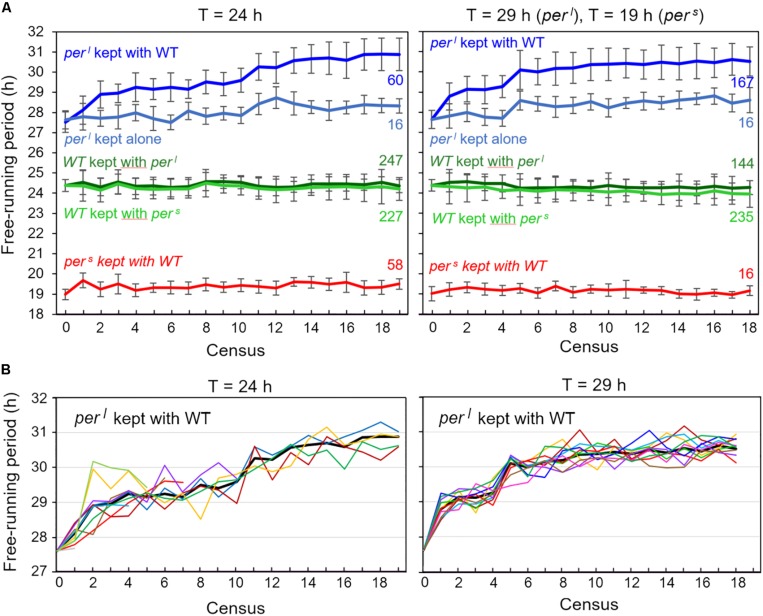
Free-running periods of the flies recorded during the two competition experiments [at Zeitgeber cycles (T) of 24 h (left) and *T* = 29 h or *T* = 19 h (right)]. Every census, 32 flies of each of the 10 vials with the competing genotypes (*per*^*l*^/WT, *per*^*s*^/WT) were recorded in DD (= 4 × 320 flies per census, see Figure 2) and the flies‘ free-running periods determined and plotted (mean of all flies from all 10 vials ± SD) **(A)**. The experiment started with equal numbers (∼160 at census 1) for each genotype and ended with a lower number of mutants, except for *per*^*l*^ mutants kept under *T* = 29 h (right diagram). The numbers at the right margin give the number of individuals for each genotype at the last census (19 under *T* = 24 h and 18 under *T* = 19/29 h). In case of *per*^*s*^ the flies included in the determination of period stem only from few vials (3 under *T* = 24 h, and 1 under *T* = 19 h). In case of *per*^*l*^, the number of vials was larger (4 under *T* = 24 h, and 10 under *T* = 29 h). Wildtype flies were present in all 10 vials, except for the competition experiment with *per*^*l*^ mutants under *T* = 29 h. Here the calculated periods at census 18 stem from flies in 8 vials. Note that the sum of mutants and WT flies is always lower than 320, because some flies died during the recording. The pale blue curve give the mean period of *per*^*l*^ mutants that were kept separately (not in competition with wildtype flies under the same environmental conditions as the experimental animals) and that were recorded in parallel to the other flies (16 flies per census). While the *per*^*l*^ mutants grown in competition with WT flies lengthened their period by ∼3 h, the ones kept separately did so only by <1 h **(B)**. Period lengthening of *per*^*l*^ mutants over the course of the competition studies in the single fly vials at *T* = 24 h and *T* = 29 h. The mean free-running periods of the flies for each of the 10 vials are shown as colored lines. The thick black line shows the average period of all flies that is also depicted in **A**. At *T* = 24 h, *per*^*l*^ mutants persisted only in 4 vials until the end of the experiment. Therefore, six colored lines ended before census 19. This is different at *T* = 29 h, where *per*^*l*^ mutants persisted in all 10 fly vials (compare Figure 7).

In the original paper (Konopka and Benzer, 1971), *per*^*s*^ mutants were reported to free-run with a period of ∼19 h and *per*^*l*^ mutants with a period of ∼ 28 h under constant darkness. We determined the free-running period and the activity patterns under LD12:12 for 60 wildtype and mutant flies, respectively, before they underwent the competition experiment. We found a mean period of 24.3 ± 0.04 h for wildtype flies, a period of 19.0 ± 0.03 h for *per*^*s*^ mutants and a period of 27.6 ± 0.06 h for *per*^*l*^ mutants. Thus, while the free-running period of *per*^*s*^ mutants matched the one previously reported, the free-running period of our *per*^*l*^ mutants was about 0.4 h shorter than originally reported and only 3.3 h longer than that of wildtype flies. To see whether the periods remained the same during the competition experiments, we determined them in the flies that were recorded every census throughout the competition experiment. The results are plotted in Figure 9. To our surprise, we found that *per*^*l*^ mutants lengthened their period continuously, at the beginning very fast (at census 4 they reached already 29 h) and later slower. At the end they reached a period of 31.1 ± 0.87 h (± SD) in the *T* = 24 h experiment and a period of 30.5 ± 0.71 h (± SD) in the *T* = 29 h experiment. This period lengthening was similar in all vials (Figure 9B), indicating that it was not due to founder effects or genetic drift. The other fly strains (wildtype flies and *per*^*s*^ mutants) kept their period rather constant throughout the different experiments (Figure 9). The same was true for the wildtype flies and *per*^*s*^ mutants that were kept alone, but maintained and recorded in parallel to the competing flies (data not shown). Again, the situation was a bit different for the *per*^*l*^ mutants (pale blue line in Figure 9). *per*^*l*^ mutants kept alone, without competition with the wildtype flies did also lengthen their period over time but this lengthening was far less than for the flies kept in competition with wildtype flies: *per*^*l*^ mutants reached a maximal period of 28.7 ± 0.57 h (±SD) in the first experiment (*T* = 24 h) and of 28.6 ± 0.62 h (±SD) in the second experiment (*T* = 29 h). This shows that a period lengthening of ∼2 h was caused by growing *per*^*l*^ flies together with wildtype flies.

### Experiments Done to Reveal Putative Reasons for the Different Fitness of the Mutants

#### Differences in Sperm Number

**FIGURE 10:**
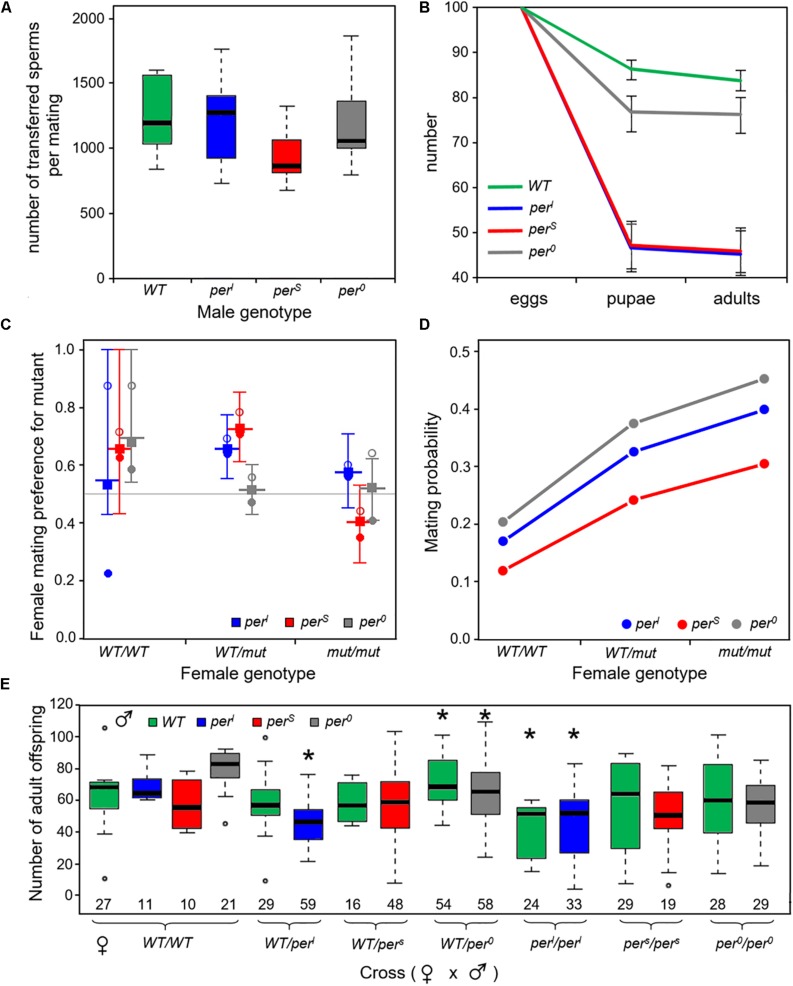
Number of transferred sperms, flies developing from 100 eggs, mating preferences of female flies and offspring of a female fly **(A)**. Mean number of transferred sperm lies between around 950 and 1250 sperms (*n* = 7 or 8 per genotype) and was not significantly different between the different fly strains **(B)**. Number of developing pupae and adults from 100 eggs of homozygous wildtype and *period* mutant flies, respectively, showing that the mutants had significant lower developmental survival rates than the wildtype flies. Error bars provide binomial 95% confidence intervals. Separate statistical analysis and significance testing was carried out from egg to pupae and then from pupae to adults (details see text) **(C)**. Estimated female mating preferences (probability to favor a mutant male over a wildtype male). The different genotypes are color coded. Open circles reflect choice in experiments where wildtype males were marked, dots in experiments were the mutant male was marked (see section“Materials and Methods”). The square shows the preference for mutant males estimated from the empirical data according to Eqs 1–5 from the main text. The longer horizontal line provides the median value and the error bars span the 95% confidence limits based on the Monte-Carlo simulations (100,000 replicates; see section “Materials and Methods”). Heterozygous *WT/per^*l*^* and *WT/per^*s*^* females preferred the mutant male, respectively. Similarly, wildtype females had a slight preference for *per*^*s*^ and *per*^0^ males (for details see text) **(D)**. Overall probability of successful mating in the mating preference tests dependent of female genotype [color code as in **(C)**]. Logistic regression indicates a significant effect of both, the female’s genotype and the mutant male under investigation. Mutant females had a higher probability to mate than wildtype females and *per*^0^ and *per*^*l*^ males were more successful in mating than *per*^*s*^ males (for details, see text) **(E)**. Number of adult offspring from the mated females shown in **(C)**. The data showed a large variability, but nevertheless revealed that *per*^*l*^ females had a lower and *WT/per^0^* a higher number of offspring (asterisks). Details see text. The number of females contributing to this analysis is indicated at the bottom of each box plot.

To test whether a reduced fertility of the males contributes to the poor performance of the *period* mutants as was shown in a previous study (Beaver et al., 2002), we counted the number of sperms that were transferred during a successful mating for the different genotypes (Figure 10A). We found no evidence for a significant difference in sperm numbers between the different genotypes (ANOVA, *F* = 1.438, df 3,27, *p* > 0.05). Wildtype males transferred on average 1257 sperms whereas males of *per*^*S*^ mutants transferred 949 sperms, about a quarter less than wildtype males. However, a female can only store about 500 sperms (Miller and Pitnick, 2002; Manier et al., 2010) and produces less than 200 offspring (see Figure 10E), so that all males supplied enough sperm to make it unlikely that the fertility of females is limited by sperm availability.

#### Differences in Survival During Development

The poor performance of the *period* mutants in the competition experiment may also be due to a lower survival of larvae during development. To test this, we counted the number of pupae and adults that emerged from 1000 eggs for each genotype. We revealed a loss of individuals during larval development in all genotypes that was clearly more dramatic in the *period* mutants as compared to the wildtype flies (Figure 10B). The statistical comparison (general linear model with quasibinomial error structure and Dunnett’s correction for *post hoc* testing) revealed that the egg to pupal survival rate (*s*_*e*_) was significantly higher for wildtype females ($s_{e}^{W\,T}=0.863$) compared to any of the three homozygous mutant females: *per*^*l*^ ($s_{e}^{p\,e\,r\,L}=0.466$, d.f. 1, 18, dev. reduction = −371.39, *F* = 110.62, *p_*adj*_* < 0.001), *per*^*s*^ ($s_{e}^{p\,e\,r\,S}=0.472$, *d.f.* 1, 18, dev. reduction = −361.62, *F* = 165.2 *p_*adj*_* < 0.001) and *per*^0^ ($s_{e}^{p\,e\,r\,0}=0.768$, *d.f.* 1, 18, dev. reduction = −30.272, *F* = 13.774, *p_*adj*_* < 0.01). Survival of *per*^0^ mutants was also significantly larger than that of the other two mutants (*p_*adj*_* < 0.001).

Survival from the pupal stage to adult (s_*p*_) was very high in all genotypes with no significant difference (GLM with binomial error structure and Dunnett’s *post hoc* correction) between wildtype flies, *per*^*l*^ and *per*^*s*^ mutants ($s_{p}^{W\,T}=0.971,s_{p}^{p\,e\,r\,L}=0.968,s_{p}^{p\,e\,r\,S}=0.971$, χ^2^ test likelihood ratio test, *p_*adj*_* > 0.9 for both comparisons). However, survivorship of *per*^0^ pupae to adult was even higher than that of wildtype pupae ($s_{p}^{p\,e\,r\,0}=0.992$, d.f. 1, 18, dev. reduction = 10.588, *p_*adj*_* < 0.05; Dunnett’s *post hoc* correction).

The results provide clear evidence for a significant effect of the clock mutants on a possible fitness component and can explain the poor performance of *per*^*s*^ mutants in the competition assay under the 24 h-day. However, they cannot explain why *per*^*l*^ mutants performed much better during the competition than the other two mutants. They can also not explain, why *per*^0^ mutants, which had the best survival among the three mutants, lost very fast and completely in the competition experiments.

#### Differences in Female Mating Preferences and Overall Mating Success

To investigate whether the persistence of *per*^*s*^ and especially *per*^*l*^ mutants in the population is due to the preferential selection of mutant males as mating partners by female flies (=rare male advantage), we determined the mating preference of females. It is known that the *period* gene affects not only daily and circadian behavior, but also the frequency of the male courtship song, which in turn strongly affects the mating willingness of females (Kyriacou et al., 1990b, 2017). Females usually prefer males that sing in the frequency of their own genotype, meaning that *per*^*s*^ females prefer *per*^*s*^ males, *per*^*l*^ females *per*^*l*^ males and wild-type females wild-type males (Greenacre et al., 1993). The mating preference of females that were heterozygous for one of the *period* mutations was, however, not tested before. Since we expect most females in our populations to be homozygous or heterozygous wildtypes, their mating preferences could clearly influence the persistence of certain mutations in the population. Therefore, we let each of 280 heterozygous females and 140 homozygous females choose between two males, a wildtype and a mutant one. We tested twice the number of heterozygous females as we wanted to account for possible effects of whether the mutant allele was inherited from the female’s mother or father; however, we could not find any difference and thus pooled the data for further analyses. Based on the creation of the Monte-Carlo confidence intervals (see Methods) we found that heterozygous *WT/per^*s*^* and *WT/per^*l*^* females significantly favored *per*^*s*^ and *per*^*l*^ males over wildtype males, respectively (Figure 10C, *p* < 0.05). In our assay, even homozygous wildtype females showed a tendency to prefer mutant males, but this was only significant for *per*^0^ males (Figure 6C, *p* < 0.05). In conclusion, the female mating preference might be a possible reason for the persistence of the mutants in the population as it would allow the mutant alleles to persist at low frequency if negative effects of the mutant gene primarily show up in homozygous individuals.

The mating preference experiments also provided information on the fraction of females that mated successfully during the 2 monitored hours (Figure 10D). Mating success ranged between 10% and 45% and was very consistently influenced by the genotype: homozygous wildtype females mated with lower probability than heterozygous females and in particular homozygous mutant females (d.f. = 2, dev. reduction = 56.605, *F* = 16.18, *p* < 0.001). In addition, mating success also depended on the male genotype (d.f. = 1, dev. reduction = 22.571, *F* = 6.45, *p* < 0.05), with the highest success when a *per*^0^ or *per*^*l*^ mutant male was among the potential mating partners. Since overall mating success depends not only on the female choice but also on the male’s courting activity there are several possible explanations for these results. Either the mutant females were less choosy than wildtype females, possibly because of their generally reduced fitness, or, for unknown reasons, they were more attractive for males. In addition, we assume that male activity affects mating success. Mating success in trios including a *per*^*l*^ male was generally higher than in trios including a *per*^*s*^ male, which fits to the high morning activity of *per*^*l*^ males as compared to that of *per*^*s*^ males (Figure 4C). However, *per*^0^ males had the highest mating success, which cannot be explained by male activity alone.

In summary, the higher mating success of mutant flies together with the preference of heterozygous females for mutant males may explain why *per*^*l*^ and *per*^*s*^ mutants persisted at low frequency in the population when grown in competition with wildtype flies. However, both observations can again not explain why *per*^0^ mutants apparently disappeared completely. Without investigating in more detail the behavioral mechanisms underlying female choice and male courtship activity we cannot be sure about underlying reasons explaining these results. One possibility is that females generally prefer to mate with “different” males providing a selective benefit for the rare males, but mating behavior and female choice may also fundamentally differ between original (highly inbred) populations.

#### Differences in the Number of Offspring per Female

After having successfully mated in the female mating preference test, the females were used to evaluate the net number of their offspring (=net fertility, Figure 10E). The data showed a large over-dispersion (variation) requiring a “quasipoisson” error structure in the general linear model used for the statistical analysis (see materials and methods). Typically, such over-dispersion indicates that some important explanatory variables such as temperature, light, food supply (quality and amount) were not included into the statistical model. All these factors have been tightly controlled, but nevertheless, we observed that sometimes the food dried to a different degree slowing down the development of the larvae. This might explain the large between vial variance observed in the data.

In spite of the large variance, the statistical analysis revealed an effect of the mutant allele via the female genotype for *per*^*l*^ and *per*^0^ but no effect of the mating partner’s genotype (*per*^*l*^ d.f. 2, 104, dev. reduction = 93.926, *F* = 6.83, *p* < 0.01; *per*^0^: d.f. 2, 159, dev. reduction = 63.716, *F* = 4.8032 *p* < 0.01). *Posthoc* testing with Bonferroni correction showed that both the WT/*per*^*l*^ and the *per*^*l*^/*per*^*l*^ females were significantly less fertile than homozygous wildtype flies (*p_*adj*_* < 0.05), while WT/*per*^0^ females had a higher fertility than homozygous *per*^0^/*per*^0^ females (*p_*adj*_* < 0.05). The other pairwise comparisons revealed no significant differences. For *per*^*s*^ mutants we could not find any evidence for an effect of the mutant on fertility. For *per*^*l*^ mutants these results are in line with the developmental survival analysis (Figure 10B), in which the number of flies developing from 100 eggs was tested. In both experiments, *per*^*l*^ mutants showed significantly lower fitness parameters. For *per*^*s*^ and *per*^0^ mutants this was different. Although significantly less flies developed from 1000 eggs in comparison to wildtype flies (Figure 10B), a single female mutant fly had the same number of offspring or in case of WT/*per*^0^ heterozygotes even more offspring (Figure 10E). Possibly, the female mutants had laid more eggs. It is also possible that the genotype of the larvae affected survival. Since we have not genotyped the offspring in our fertility test, we do not know whether a different mortality risk of the larval genotype has contributed to the number of offspring.

In summary, we conclude that multiple factors contribute to the fitness of the *period* mutant flies. There was little effect of the genotype on sperm number, but the *per*^*l*^ mutants had clearly less offspring when kept without competition. On the other hand, *per*^*s*^ and *per*^*l*^ mutants were preferred as mating partners by heterozygous females and the mating success was larger with mutant females, which might explain why the mutant alleles remained in the population. We could not identify any reason explaining why *per*^0^ mutants disappeared rather quickly in the population when grown under competition, as the performance of the *per*^0^ mutants was better than that of the other two mutants in all tested fitness components. We acknowledge that there are many more factors that may contribute to genotype distribution in the competition assays that we have not investigated here. For example, competition could have affected developmental time differently in the genotypes, or the genotypes could have responded differently to larval crowding.

## Discussion

We show here - for the first time for an animal - that the circadian clock confers a significant competitive fitness advantage under laboratory and semi-natural outdoor conditions. In the case of *per*^*l*^ mutants, we also show that, this selective advantage depends on the resonance between the endogenous period of the animals and the period of the Zeitgeber cycle. Thus, for *per*^*l*^ mutants grown in competition with wild-type flies the resonance hypothesis is partly confirmed: proper timing (=optimal phase of the rhythm in relation to the environmental cycle) appears of significant advantage for the reproductive success of flies that compete with others.

In contrast, *per*^*s*^ mutants did not outcompete wild-type flies when grown under 19-h days, although the 19 h cycle matches their endogenous period. Furthermore, *per*^0^ mutants are not equally fit as wild-type flies under LL conditions despite the fact that LL makes wild-type flies equally arrhythmic as *per*^0^ so that they should lose all timing-advantages over the mutants. These results clearly indicate that other factors besides timing contribute to the competitive fitness benefit of wild-type flies.

It is known that the *period* gene has pleiotropic effects on fly behavior, development and fecundity. Besides affecting the frequency of the male courtship song (Kyriacou et al., 1990b, 2017), it affects developmental timing (Kyriacou et al., 1990a), sleep length (Shaw et al., 2002), the activity of neurons involved in fast escape responses (Megighian et al., 2001), the fecundity of male and female fruit flies (Beaver et al., 2002, 2003) and the duration of the copulation (Beaver and Giebultowicz, 2004). All this may negatively affect the fitness of the mutants under the 24-h day, but it is not clear why *per*^*l*^ mutants had the best and *per*^0^ the worst performance in the long-term competition experiments despite the fact that *per*^0^ performed better on any of the direct fitness estimators investigated than the two other *period* mutants. Putative reasons will be discussed in the following paragraphs. It is important to note here that all experiments addressing fitness parameters in the *period* mutants, including the ones performed here, have been undertaken under a 24-h day. Therefore, in the strict sense, our discussion applies only to the 24-h day and not to the T-cycle experiments.

### *Per ^*l*^* Mutants Appear to Have a Higher Fitness Than the Other Period Mutants

In the 24-h day, *per*^*l*^ mutants remained at ∼20% in the fly population and in one food vial they even dominated over the wildtype flies (Figure 7). The main reason for their relative higher competitive fitness, may lie in their longer endogenous period that enabled them to have a long and pronounced morning activity (Figure 4). Since courting and mating occurs mainly in the morning (Sakai and Ishida, 2001a, b), *per*^*l*^ males might be more successful in mating with females simply because they are rather active in the morning hours. It might be irrelevant for their competitive fitness when they afterward extend the siesta until the evening and start eating only in the night. Indeed, we found that *per*^*l*^ mutants had a relatively high mating success in the female mating preference assay (Figure 10D). Another positive factor for their fitness may be the mating preference of virgin *WT_CS_/per^*l*^* heterozygotes for *per*^*l*^ males (Figure 10C). These positive effects may have dominated over the lower survival rate of *per*^*l*^ larvae (Figure 10B) and the reduced number of adult offspring of *per*^*l*^ females (Figure 10E); note also that we only have egg-to-adult survival data only for homozygous individuals so that we do not know how strong the survival effect of *period* alleles is in heterozygous individuals. Earlier studies even reported a slower development of *per*^*l*^ mutants (Kyriacou et al., 1990a). In our study, we did not observe evident differences in developmental timing, but we cannot exclude that minor differences exist, especially not under competition. In any case, such differences had obviously no negative effects on the competitive fitness of *per*^*l*^ mutants.

### *Per ^*s*^* Mutants Are Clearly Less Fit Than Wild-Type Flies in the 24-h and 19-h Day, but Nevertheless Persist in the Population

*per*^*s*^ mutants persist in the population at ∼10%, independently of the external Zeitgeber period. Putative reasons for their persistence may be the strong preference of *WT/per^*s*^* heterozygote females and even of homozygote wildtype females for *per*^*s*^ males. In particular, when the mutant allele becomes rare and thus hardly occurs in homozygous females, the mutant male benefit may compensate for the female fitness disadvantages thus resulting in a stable coexistence of wild-type and mutant alleles.

Perhaps more difficult to explain is the very low overall fitness of *per*^*s*^ mutants in comparison to wildtype flies. *per*^*s*^ males transfer slightly less number of sperm (Figure 10A), but as explained earlier this is unlikely to affect the reproductive fitness of the females. The low number of surviving *per*^*s*^ larvae is for sure reducing the fitness of *per*^*s*^ mutants and in contrast to *per*^*l*^ mutants this is not compensated by a higher mating success of *per*^*s*^ males (Figure 10D). Nevertheless, the *per*^*s*^ genotype had no negative effect on the number of adult offspring (Figure 10E), suggesting that *per*^*s*^ female may compensate the high larval mortality by laying more eggs. Once again, the most likely reason for the low fitness of *per*^*s*^ mutants may lie in the activity pattern of *per*^*s*^ males (Figure 4A). In contrast to *per*^*l*^ mutants and wildtype flies, *per*^*s*^ mutants are rather inactive during the morning. Before lights-on, their activity appears suppressed by darkness and after lights-on they show only a very brief morning activity before they enter the siesta. Consequently, the mating activity of male flies may be rather low and this fits with their rather low mating success (Figure 10D). Most interestingly, this may also explain why *per*^*s*^ mutants do not perform better under the 19-h day, because even then the morning activity of male mutants was lower than that of wildtype males (Figure 6). Nevertheless, still other factors that are independent of timing may contribute to the low fitness of *per*^*s*^ mutants.

### *Per*^0^ Mutants Lose Completely Against Wild-Type Flies Under Both Tested Conditions

*per*^0^ mutants appear to have no fitness advantage against wild-type flies. The initial decline in the first generations was in fact somewhat slower than that for the other two mutants (cf. Figure 7) but did not stabilize at low proportion as seems to be the case for other two mutants. They even lose quickly against wildtype flies under LL conditions, under which the wildtype flies are similarly arrhythmic and can therefore take no advantage of their circadian clock. These findings are hard to explain in light of the experiments performed in this study. In contrast to previous observations (Greenacre et al., 1993; Beaver et al., 2002), we found no reduction of transferred sperm in *per*^0^ mutants (Figure 10A). Furthermore, the survival of *per*^0^ from egg to pupa was much better than that of *per*^*l*^ and *per*^*s*^ mutants (Figure 10B) and homozygote wildtype flies showed a mating preference for *per*^0^ males (Figure 10C). The mating success of *per*^0^ males was also very good (Figure 10D) and heterozygous WT/*per*^0^ mutants showed even a tendency to have more adult offspring than wildtype flies (Figure 10E). Consequently, there must exist other factors that reduce the fitness of *per*^0^ mutants and that possible only contribute when the flies have to compete with wildtype flies. *per*^0^ mutants are reported to sleep less (Shaw et al., 2002), to have deficits in certain aspects of learning and memory (Chouhan et al., 2015), to show a lower neuronal activity in neurons that control fast escape responses (Megighian et al., 2001), and to be more sensitive to oxidative stress (Krishnan et al., 2008). Constant light (especially short-wavelength light) also generates stress, and this might be one reason why *per*^0^ flies have disadvantages against wildtype flies under such conditions. Altogether, this might explain the observed reduced reproductive fitness of *per*^0^ mutants when raised in competition to wildtype flies. To sort this out in more detail, additional experiments are needed, which also decipher the effects of population density on the mutant’s fitness under competition conditions.

### Period Lengthening of *per ^*l*^* Mutants and Putative Effects of the Genetic Background

One of the most surprising result of our study was the dramatic period lengthening of the *per*^*l*^ mutants over the course of the competition experiment. At the beginning, the mutants had slightly shorter periods than reported in previous studies (Konopka and Benzer, 1971), but already at census 2 (generation 6) they reached periods of ∼29 h, and then asymptotically approached periods of 31 h in both experiments (under the 24 h and the 29 h day; Figure 9). A selection for long periods by the 29 h cycle cannot be the cause of this period lengthening, because it occurred also under the 24 h cycle. Most interestingly, the 31 h period precisely coincides with the period that Ewer et al. (1990) measured in *per*^*l*^ mutants at 25°C. Possibly, during the long inbreeding, our *per*^*l*^ mutants have accumulated genetic modifiers that shortened their period so that it stayed closer to 24 h. This would imply that wildtype and mutant flies had not the identical genetic background and were probably not outcrossed with each other at the beginning of the experiment as we had presumed. During the gradual exchange of the genetic background between wildtype and *per*^*l*^ mutants in the competition experiment, the genetic modifiers may have got lost and the original period of *per*^*l*^ mutants has reappeared. This interpretation is supported by the fact that *per*^*l*^ kept in isolation do not show a comparable lengthening of their period. In wildtype flies and *per*^*s*^ mutants, we could not see any evident changes in period during the competition experiment. However, this does not mean that these flies had an identical genetic background. Future studies with the now perfectly “cantonized” *period* mutants are necessary to clarify this.

### Conclusion

We have shown that possessing an endogenous clock that runs with a period close to 24 h has a clear fitness advantage when flies have to compete with others for food and mating partners. The right timing of activity, especially in the morning when courting and mating occurs, may be crucial for the survival in the population. In addition, we have shown that multiple other factors contribute to the performance of the mutants. Especially the *per*^*s*^ and *per*^0^ mutants are clearly less fit as compared to wildtype flies. This confirms previous observations that the *period* gene has pleiotrophic effects on physiology, metabolism and behavior, independent of its effect on timing. Furthermore, we have clear evidences that the genetic background contributes, too. At least *per*^*l*^ mutants appeared to have accumulated background mutations that modulated the effects of the *per*^*l*^ mutation on the circadian period in such a way that it remained closer to 24 h than in the original stocks maintained at 24 h cycles. After now having outcrossed the flies for >50 generations, we have certainly eliminated such background differences. Thus, we are in the perfect situation to test the impact of the *period* gene on fitness in more detail in future experiments.

## Data Availability Statement

The raw data supporting the conclusion of this manuscript will be made available by the authors, without undue reservation, to any qualified researcher.

## Author Contributions

MH performed the experiments. OM and TH analyzed the data statistically, made predictions, and contributed to planning of the experiments. TY, DR, and CH-F conceived the study and planned the experiments. DR supervised the experiments. CH-F wrote the manuscript with contributions by DR and TH.

## Conflict of Interest

The authors declare that the research was conducted in the absence of any commercial or financial relationships that could be construed as a potential conflict of interest.

## Footnotes

1 https://fiji.sc/

## References

[B1] Abhilash L. Sharma V. K. (2016). On the relevance of using laboratory selection to study the adaptive value of circadian clocks. *Physiol. Entomol.* 41 293–306. 10.1111/phen.12158

[B2] Albrecht U. Zheng B. Larkin D. Sun Z. S. Lee C. C. (2001). MPer1 and mper2 are essential for normal resetting of the circadian clock. *J. Biol. Rhythms* 16 100–104. 10.1177/074873001129001791 11302552

[B3] Beaver L. M. Giebultowicz J. M. (2004). Regulation of copulation duration by period and timeless in *Drosophila melanogaster*. *Curr. Biol.* 14 1492–1497. 10.1016/j.cub.2004.08.022 15324667

[B4] Beaver L. M. Gvakharia B. O. Vollintine T. S. Hege D. M. Stanewsky R. Giebultowicz J. M. (2002). Loss of circadian clock function decreases reproductive fitness in males of *Drosophila melanogaster*. *Proc. Natl. Acad. Sci. U.S.A.* 99 2134–2139. 10.1073/pnas.032426699 11854509 PMC122331

[B5] Beaver L. M. Rush B. L. Gvakharia B. O. Giebultowicz J. M. (2003). Noncircadian regulation and function of clock genes period and timeless in oogenesis of *Drosophila melanogaster*. *J. Biol. Rhythms* 18 463–472. 10.1177/0748730403259108 14667147

[B6] Chouhan N. S. Wolf R. Helfrich-Förster C. Heisenberg M. (2015). Flies remember the time of day. *Curr. Biol.* 25 1619–1624. 10.1016/j.cub.2015.04.032 26028434

[B7] Crawley M. J. (2013). *The R Book*, 2nd Edn. Chichester: Wiley.

[B8] Daan S. Spoelstra K. Albrecht U. Schmutz I. Daan M. Daan B. (2011). Lab mice in the field: unorthodox daily activity and effects of a dysfunctional circadian clock allele. *J. Biol. Rhythms* 26 118–129. 10.1177/0748730410397645 21454292

[B9] DeCoursey P. J. (2014). Survival value of suprachiasmatic nuclei (SCN) in four wild sciurid rodents. *Behav. Neurosci.* 128 240–249. 10.1037/a0036696 24886187

[B10] DeCoursey P. J. Krulas J. R. (1998). Behavior of SCN-lesioned chipmunks in natural habitat: a pilot study. *J. Biol. Rhythms* 13 229–244. 10.1177/074873098129000075 9615287

[B11] DeCoursey P. J. Walker J. K. Smith S. A. (2000). A circadian pacemaker in free-living chipmunks: essential for survival? *J. Comp. Physiol. A* 186 169–180. 10.1007/s003590050017 10707315

[B12] Dodd A. N. Salathia N. Hall A. Kévei E. Tóth R. Nagy F. (2005). Plant circadian clocks increase photosynthesis, growth, survival, and competitive advantage. *Science* 309 630–633. 10.1126/science.1115581 16040710

[B13] Ewer J. Hamblen-Coyle M. Rosbash M. Hall J. C. (1990). Requirement for period gene expression in the adult and not during development for locomotor activity rhythms of imaginal *Drosophila melanogaster*. *J. Neurogenet.* 7 31–73. 10.3109/01677069009084151 2129172

[B14] Garbaczewska M. Billeter J.-C. Levine J. D. (2013). *Drosophila melanogaster* males increase the number of sperm in their ejaculate when perceiving rival males. *J. Insect Physiol.* 59 306–310. 10.1016/j.jinsphys.2012.08.016 23178803

[B15] Greenacre M. L. Ritchie M. G. Byrne B. C. Kyriacou C. P. (1993). Female song preference and the period gene in *Drosophila*. *Behav. Genet.* 23 85–90. 10.1007/bf01067557 8476395

[B16] Hamblen M. J. White N. E. Emery P. T. Kaiser K. Hall J. C. (1998). Molecular and behavioral analysis of four period mutants in *Drosophila melanogaster* encompassing extreme short, novel long, and unorthodox arrhythmic types. *Genetics* 149 165–178. 9584094 10.1093/genetics/149.1.165 PMC1460118

[B17] Hamblen-Coyle M. J. Wheeler D. A. Rutila J. E. Rosbash M. Hall J. C. (1992). Behavior of period-altered circadian rhythm mutants of *Drosophila* in light: dark cycles (Diptera: Drosophilidae). *J. Insect Behav.* 5 417–446. 10.1007/BF01058189

[B18] Johnson C. H. Mori T. Xu Y. (2008). A cyanobacterial circadian clockwork. *Curr. Biol.* 18 R816–R825. 10.1016/j.cub.2008.07.012 18786387 PMC2585598

[B19] Klarsfeld A. Rouyer F. (1998). Effects of circadian mutations and LD periodicity on the life span of *Drosophila melanogaster*. *J. Biol. Rhythms* 13 471–478. 10.1177/074873098129000309 9850008

[B20] Konopka R. J. Benzer S. (1971). Clock mutants of *Drosophila melanogaster*. *Proc. Natl. Acad. Sci. U.S.A.* 68 2112–2116. 10.1073/pnas.68.9.2112 5002428 PMC389363

[B21] Krishnan N. Davis A. J. Giebultowicz J. M. (2008). Circadian regulation of response to oxidative stress in *Drosophila melanogaster*. *Biochem. Biophys. Res. Commun.* 374 299–303. 10.1016/j.bbrc.2008.07.011 18627767 PMC2553425

[B22] Kyriacou C. P. Green E. W. Piffer A. Dowse H. B. (2017). Failure to reproduce period-dependent song cycles in *Drosophila* is due to poor automated pulse-detection and low-intensity courtship. *Proc. Natl. Acad. Sci. U.S.A.* 114 1970–1975. 10.1073/pnas.1615198114 28174268 PMC5338454

[B23] Kyriacou C. P. Oldroyd M. Wood J. Sharp M. Hill M. (1990a). Clock mutations alter developmental timing in *Drosophila*. *Heredity* 64(Pt 3), 395–401. 10.1038/hdy.1990.50 2113515

[B24] Kyriacou C. P. van den Berg M. J. Hall J. C. (1990b). *Drosophila* courtship song cycles in normal and period mutant males revisited. *Behav. Genet.* 20 617–644. 10.1007/bf01065875 2126924

[B25] Lenth R. (2019). *Emmeans: Estimated Marginal Means, aka Least-Squares Means. R Package Version 1.4.* Available at: https://CRAN.R-project.org/package=emmeans

[B26] Manier M. K. Belote J. M. Berben K. S. Novikov D. Stuart W. T. Pitnick S. (2010). Resolving mechanisms of competitive fertilization success in *Drosophila melanogaster*. *Science* 328 354–357. 10.1126/science.1187096 20299550

[B27] Megighian A. Zordan M. Costa R. (2001). Giant neuron pathway neurophysiological activity in per(0) mutants of *Drosophila melanogaster*. *J. Neurogenet.* 15 221–231. 10.3109/01677060109167378 12092905

[B28] Mendoza J. Albrecht U. Challet E. (2010). Behavioural food anticipation in clock genes deficient mice: confirming old phenotypes, describing new phenotypes. *Genes Brain Behav.* 9 467–477. 10.1111/j.1601-183X.2010.00576.x 20180860

[B29] Miller G. T. Pitnick S. (2002). Sperm-female coevolution in *Drosophila*. *Science* 298 1230–1233. 10.1126/science.1076968 12424377

[B30] Nikhil K. Sharma V. K. (2017). “On the origin and implications of circadian time-keeping: an evolutionary perspective,” in *Biological Timekeeping: Clocks, Rhythms and Behaviour*, ed. Kumar V., (Berlin: Springer), 81–129. 10.1007/978-81-322-3688-7_5

[B31] Ouyang Y. Andersson C. R. Kondo T. Golden S. S. Johnson C. H. (1998). Resonating circadian clocks enhance fitness in cyanobacteria. *Proc. Natl. Acad. Sci. U.S.A.* 95 8660–8664. 10.1073/pnas.95.15.8660 9671734 PMC21132

[B32] Patke A. Murphy P. J. Onat O. E. Krieger A. C. Özçelik T. Campbell S. S. (2017). Mutation of the human circadian clock gene CRY1 in familial delayed sleep phase disorder. *Cell* 169 203.e13–215.e13. 10.1016/j.cell.2017.03.027 28388406 PMC5479574

[B33] Pendergast J. S. Friday R. C. Yamazaki S. (2010). Photic entrainment of period mutant mice is predicted from their phase response curves. *J. Neurosci.* 30 12179–12184. 10.1523/JNEUROSCI.2607-10.2010 20826680 PMC2943870

[B34] Pittendrigh C. S. Minis D. H. (1972). Circadian systems: longevity as a function of circadian resonance in *Drosophila melanogaster*. *Proc. Natl. Acad. Sci. U.S.A.* 69 1537–1539. 10.1073/pnas.69.6.1537 4624759 PMC426743

[B35] R Core Team (2018). *R: A Language and Environment for Statistical Computing.* Vienna: R Foundation for Statistical Computing Available at: https://www.R-project.org/

[B36] Ralph M. R. Menaker M. (1988). A mutation of the circadian system in golden hamsters. *Science* 241 1225–1227. 10.1126/science.3413487 3413487

[B37] Sakai T. Ishida N. (2001a). Circadian rhythms of female mating activity governed by clock genes in *Drosophila*. *Proc. Natl. Acad. Sci. U.S.A.* 98 9221–9225. 10.1073/pnas.151443298 11470898 PMC55401

[B38] Sakai T. Ishida N. (2001b). Time, love and species. *Neuro. Endocrinol. Lett.* 22 222–228. 11524627

[B39] Schlichting M. Helfrich-Förster C. (2015). Photic entrainment in *Drosophila* assessed by locomotor activity recordings. *Meth. Enzymol.* 552 105–123. 10.1016/bs.mie.2014.10.017 25707274

[B40] Schlichting M. Menegazzi P. Helfrich-Förster C. (2015). Normal vision can compensate for the loss of the circadian clock. *Proc. Biol. Sci. U.S.A.* 282:20151846. 10.1098/rspb.2015.1846 26378222 PMC4614763

[B41] Schmid B. Helfrich-Förster C. Yoshii T. (2011). A new imagej plug-in “actogramj” for chronobiological analyses. *J. Biol. Rhythms* 26 464–467. 10.1177/0748730411414264 21921300

[B42] Shaw P. J. Tononi G. Greenspan R. J. Robinson D. F. (2002). Stress response genes protect against lethal effects of sleep deprivation in *Drosophila*. *Nature* 417 287–291. 10.1038/417287a 12015603

[B43] Sheeba V. Sharma V. K. Shubha K. Chandrashekaran M. K. Joshi A. (2000). The effect of different light regimes on adult life span in *Drosophila melanogaster* is partly mediated through reproductive output. *J. Biol. Rhythms* 15 380–392. 10.1177/074873000129001477 11039916

[B44] Toh K. L. Jones C. R. He Y. Eide E. J. Hinz W. A. Virshup D. M. (2001). An hPer2 phosphorylation site mutation in familial advanced sleep phase syndrome. *Science* 291 1040–1043. 10.1126/science.1057499 11232563

[B45] Vaccaro A. Birman S. Klarsfeld A. (2016). Chronic jet lag impairs startled-induced locomotion in *Drosophila*. *Exp. Gerontol.* 85 24–27. 10.1016/j.exger.2016.09.012 27639775

[B46] van der Horst G. T. Muijtjens M. Kobayashi K. Takano R. Kanno S. Takao M. (1999). Mammalian cry1 and cry2 are essential for maintenance of circadian rhythms. *Nature* 398 627–630. 10.1038/19323 10217146

[B47] Vanin S. Bhutani S. Montelli S. Menegazzi P. Green E. W. Pegoraro M. (2012). Unexpected features of *Drosophila* circadian behavioural rhythms under natural conditions. *Nature* 484 371–375. 10.1038/nature10991 22495312

[B48] Vaze K. M. Sharma V. K. (2013). On the adaptive significance of circadian clocks for their owners. *Chronobiol. Int.* 30 413–433. 10.3109/07420528.2012.754457 23452153

[B49] Wheeler D. A. Hamblen-Coyle M. J. Dushay M. S. Hall J. C. (1993). Behavior in light-dark cycles of *Drosophila* mutants that are arrhythmic, blind, or both. *J. Biol. Rhythms* 8 67–94. 10.1177/074873049300800106 8490212

